# Emerging mutation in SARS-CoV-2 facilitates escape from NK cell recognition and associates with enhanced viral fitness

**DOI:** 10.1371/journal.ppat.1012755

**Published:** 2024-12-09

**Authors:** Eleni Bilev, Nicole Wild, Pouria Momayyezi, Benedetta Maria Sala, Renhua Sun, Tatyana Sandalova, Nicole Marquardt, Hans-Gustaf Ljunggren, Adnane Achour, Quirin Hammer

**Affiliations:** 1 Center for Infectious Medicine, Department of Medicine Huddinge, Karolinska Institutet, Karolinska University Hospital Huddinge, Stockholm, Sweden; 2 Center for Hematology and Regenerative Medicine, Department of Medicine Huddinge, Karolinska Institutet, Stockholm, Sweden; 3 Science for Life Laboratory, Department of Medicine Solna, Karolinska Institutet & Division of Infectious Diseases, Karolinska University Hospital Solna, Stockholm, Sweden; Vaccine Research Center, UNITED STATES OF AMERICA

## Abstract

In addition to adaptive immunity, natural killer (NK) cells of the innate immune system contribute to the control of viral infections. The HLA-E-restricted SARS-CoV-2 Nsp13_232-240_ epitope VMPLSAPTL renders infected cells susceptible to NK cells by preventing binding to the inhibitory receptor NKG2A. Here, we report that a recently emerged methionine to isoleucine substitution at position 2 (pM2I) of Nsp13_232-240_ impairs binding of the mutated epitope to HLA-E and diminishes HLA-E/peptide complex stability. Structural analyses revealed altered occupancy of the HLA-E B-pocket as the underlying cause for reduced presentation and stability of the mutated epitope. Functionally, the reduced presentation of the mutated epitope correlated with elevated binding to NKG2A as well as with increased NK cell inhibition. Moreover, the pM2I mutation associated with enhanced estimated viral fitness and was transmitted to descendants of the SARS-CoV-2 BQ.1 variant. Interestingly, the mutated epitope resembles sequences of related peptides found in endemic common cold-causing human coronaviruses. Altogether, these findings indicate compromised peptide presentation as a viral adaptation to evade NK cell-mediated immunosurveillance by enabling enhanced presentation of self-peptide and restoring NKG2A-dependent inhibition of NK cells.

## Author summary

SARS-CoV-2 continues to evolve and adapts to immunity acquired by infection or vaccination. However, whether SARS-CoV-2 also adapts to innate immune cells such as natural killer (NK) cells remains largely unknown. Here, we found that the Nsp13_232-240_ epitope, which is recognized by NK cells, is mutated in the Omicron BQ.1 sub-variant of SARS-CoV-2. This emerging mutation results in reduced presentation of the mutated epitope and correlates with less NK cell activity. Interestingly, the mutated epitope shares characteristics with related peptides found in common cold-causing coronaviruses, which are endemic in humans. Therefore, it is conceivable that this mutation represents an adaptation of SARS-CoV-2 to escape from NK cell responses.

## Introduction

Rapid adaptation of viruses to immune pressure is a hallmark of continuous host-pathogen interactions. Accordingly, mutations associated with impaired vaccine- or infection-induced immunity mediated by the adaptive immune system have been well characterized for severe acute respiratory coronavirus 2 (SARS-CoV-2), and the continuous emergence of novel immune evasive variants has contributed to successive waves of infection [1,2]. In contrast, mutations enabling viral evasion from innate immune responses have remained comparably unexplored.

Natural killer (NK) cells are innate immune cells that contribute to the control of viral infections [3]. In line with their anti-viral functions, NK cells are robustly activated in patients with acute COVID-19 [4–7]. Moreover, high numbers of peripheral NK cells correlate with rapidly declining viral load following SARS-CoV-2 infection [6] and NK cells can suppress SARS-CoV-2 replication *in vitro* [6–8] as well as in a non-human primate model [9]. We have previously demonstrated that the non-structural protein 13 (Nsp13) of SARS-CoV-2 contains the HLA-E-restricted Nsp13_232-240_ epitope VMPLSAPTL, which hinders binding of HLA-E/peptide complexes to the inhibitory NK cell receptor NKG2A [8]. This is in stark contrast to HLA-E-restricted self-peptides, which bind NKG2A and suppress NK cell activity, thereby maintaining tolerance to self [10]. Consequently, excessive presentation of the Nsp13_232-240_ epitope without binding the NKG2A receptor reduces inhibition and facilitates activation, thereby rendering infected cells susceptible to attack by NK cells. However, it remains unclear whether SARS-CoV-2 adapts to this antagonized inhibition and recognition by NK cells.

Here, we report that a single amino acid substitution within the Nsp13_232-240_ epitope emerged in the Omicron BQ.1 sub-variant. The mutated epitope displays diminished presentation and altered occupancy in the B-pocket of HLA-E. Compared to the ancestral epitope, reduced presentation of the mutated epitope resulted in increased NK cell inhibition in settings of mixed peptide repertoires containing self-peptide as well as virus-derived peptide. Moreover, the mutated epitope is associated with enhanced estimated fitness of SARS-CoV-2, is transmitted to descendant sub-lineages, and approximates the sequences as well as the poor presentation of related peptides found in common cold-causing human coronaviruses (HCoV).

## Results

### Emergence of a mutation in the Nsp13_232-240_ epitope of the BQ.1 sub-lineage

**Fig 1:**
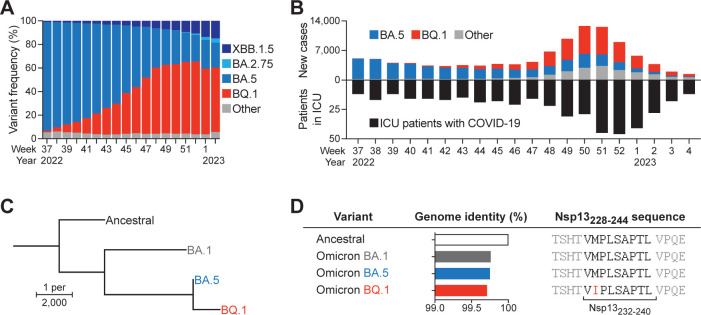
A single amino acid substitution in the Nsp13_232-240_ epitope is associated with enhanced viral fitness. (**A**) Dynamics of variant frequencies detected by genomic surveillance. Combined data reported from Belgium, France, Germany, Norway, Portugal, and Sweden. (**B**) Newly reported infection cases (upper bars) and number of patients receiving intensive care tested positive for COVID-19 (lower bars) in Sweden. Reported infection cases are colored based on percentage of variant prevalence. (**C**) Phylogenic tree illustrating genomic divergence between ancestral SARS-CoV-2 and Omicron sub-lineages as determined by Clustalω. Length of branches denotes relative divergent nucleotides. (**D**) Genomic sequence identity between ancestral SARS-CoV-2 and Omicron sub-lineages as determined by Clustalω and Nsp13_232-240_ sequences of the indicated sub-lineages.

The SARS-CoV-2 Omicron sub-lineage BQ.1 rapidly displaced its parental BA.5 sub-lineage (**Fig 1A**), and the spread of this new variant was accompanied by a local surge of infections resulting in increased numbers of infected patients requiring intensive care in Sweden (**Fig 1B**). BA.5 and BQ.1 display substantial genetic homology (**Fig 1C**) and differ in only six amino acids across the entire virus proteome. In addition to two described mutations in the Spike protein [11,12], one of the mutations corresponding to the dominance and spread of BQ.1 is a single nucleotide substitution, which results in a methionine to isoleucine substitution at position 2 (pM2I) in the HLA-E-restricted Nsp13_232-240_ epitope (**Figs 1D and S1**).

### The pM2I mutation diminishes presentation by HLA-E

**Fig 2:**
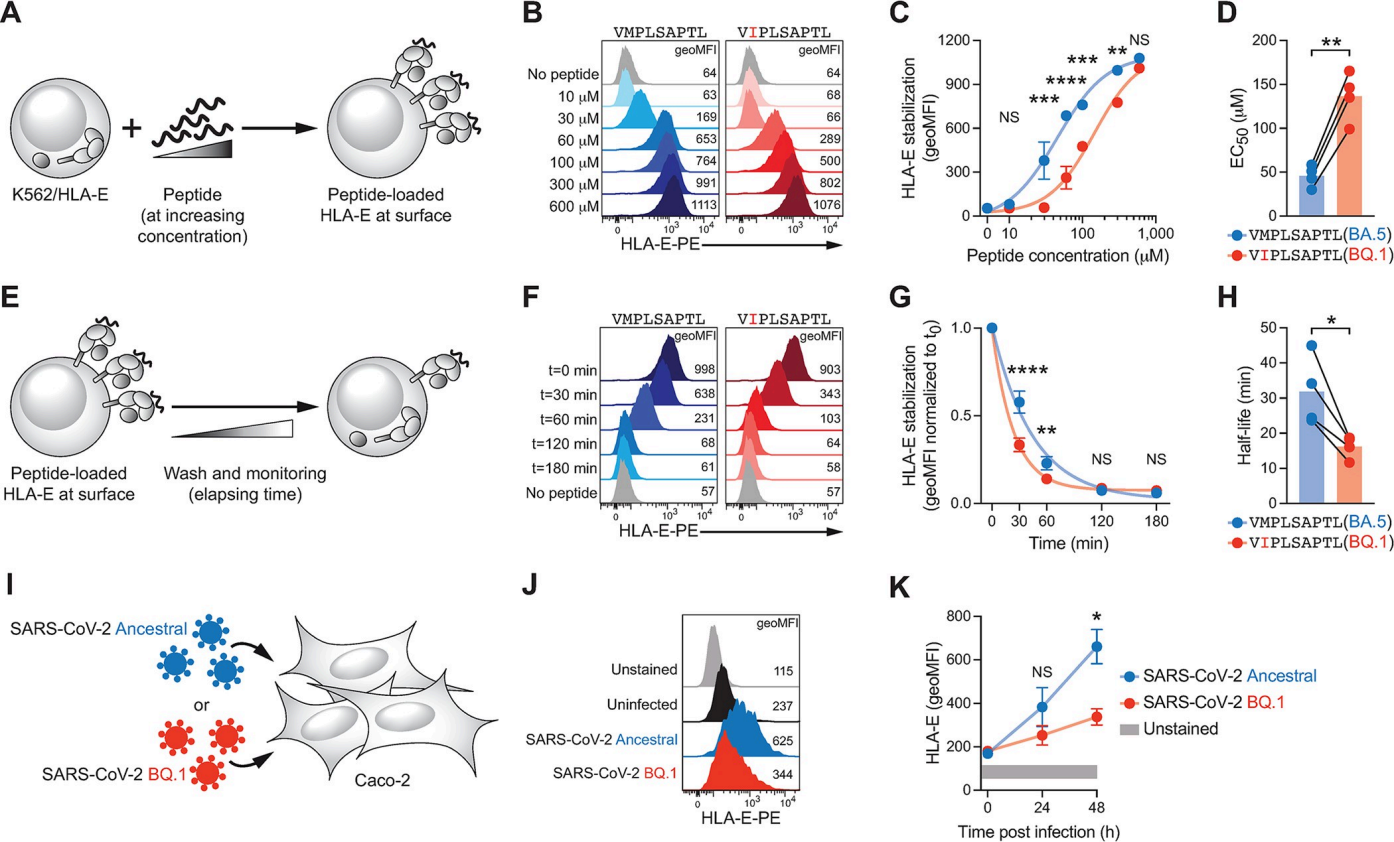
The pM2I mutation diminishes presentation efficiency. (**A**) Schematic illustration depicting cellular peptide binding assays based on loading of K562/HLA-E cells with different concentrations of synthetic peptides. (**B**) Representative cell surface HLA-E levels determined by flow cytometry upon loading with indicated concentrations of peptides. Numbers denote geometric mean fluorescence intensity (geoMFI). (**C**) Summary of HLA-E signals with dots denoting mean, error bars SEM, and lines dose-response curves (n = 4 independent experiments). Two-way ANOVA with Šídák’s multiple comparisons test (NS p ≥ 0.05, ** p <  0.01, *** p < 0.001, and **** p < 0.0001). (**D**) Concentrations required for half-maximal surface HLA-E signals (EC_50_). Connected dots denote individual experiments and bars mean (n = 4 independent experiments). Paired t-test (** p <  0.01). (**E**) Schematic illustration depicting cellular HLA-E/peptide complex turnover assays based on loading of K562/HLA-E cells with peptides and subsequent removal. (**F**) Representative cell surface HLA-E levels determined by flow cytometry after removal of peptides since indicated times. Numbers denote geoMFI. (**G**) Summary of HLA-E levels relative to t = 0 min timepoint (t_0_). Dots denote means, error bars SEM, and lines exponential decay curves (n = 4 independent experiments). Two-way ANOVA with Šídák’s multiple comparisons test (NS p ≥ 0.05, ** p <  0.01, and **** p < 0.0001). (**H**) Half-life of HLA-E/peptide complexes. Connected dots denote individual experiments and bars mean (n = 4 independent experiments). Paired t-test (* p <  0.05). (**I**) Schematic illustration depicting *in vitro* infection experiments by inoculation of Caco-2 cells with either SARS-CoV-2 Ancestral or SARS-CoV-2 BQ.1. (**J**) Representative cell surface HLA-E levels determined by flow cytometry following infection with MOI = 1. Numbers denote geoMFI. (**K**) Summary of HLA-E levels over time post-infection. Dots denote means and error bars SEM (n = 5 infections in n = 3 independent experiments). Two-way ANOVA with Šídák’s multiple comparisons test (NS p ≥ 0.05 and * p <  0.05).

The Nsp13_232-240_ epitope is presented by HLA-E [8,13] and position 2 serves as a main anchor for HLA-E-restricted peptides [14,15]. To assess the presentation efficiency of the mutated epitope, we conducted cellular HLA-E binding assays by loading exogenous peptides onto K562/HLA-E cells, which express HLA-E*01:03 but do not present endogenous peptides. We then measured the formation of HLA-E/peptide complexes on the cell surface (**Fig 2A**). Over a broad range of concentrations, BQ.1 Nsp13_232-240_ showed significantly impaired induction of HLA-E/peptide complexes compared to BA.5 Nsp13_232-240_ (**Fig 2B and 2C**). The mutated epitope further required higher concentrations for half-maximal binding to HLA-E, indicative of overall reduced affinity (**Fig 2D**). Lysine substitution scanning along the BA.5 Nsp13_232-240_ epitope confirmed the importance of position 2 for efficient HLA-E presentation, along with contributions by residues in positions 3, 6, 7, and 9 (**S2A and S2B Fig**), which is in line with previously described motif features of canonical HLA-E-restricted peptides [14–18].

As the stability of HLA/peptide complexes is a key factor correlated to their immunological function [19,20], we next performed cellular stability assays. For this, K562/HLA-E cells were loaded with peptide, after which excess peptide was removed, and surface HLA-E levels were evaluated over time (**Fig 2E**). Here, HLA-E/peptide complexes decayed more rapidly from cells loaded with the mutated BQ.1 Nsp13_232-240_ variant compared to cells loaded with the BA.5-derived epitope (**Fig 2F and 2G**). HLA-E/peptide complexes formed with BQ.1 Nsp13_232-240_ showed significantly lower half-life compared to those formed with BA.5 Nsp13_232-240_ (**Fig 2H**), further pointing towards curtailed stability.

Finally, we complemented our peptide presentation and stability assays with *in vitro* infection experiments by inoculating the epithelial cell line Caco-2 with either ancestral SARS-CoV-2 (encoding VMPLSAPTL) or SARS-CoV-2 BQ.1 (encoding VIPLSAPTL, **Fig 2I**). When monitoring HLA-E surface expression post infection, we found that the SARS-CoV-2 BQ.1 variant induced lower HLA-E levels compared to ancestral SARS-CoV-2 (**Fig 2J and 2K**). Similar results were observed upon infection with a higher viral dose and in A549-hACE2 cells (**S2C and S2D Fig**).

### The pM2I mutation alters HLA-E B-pocket occupancy

**Fig 3:**
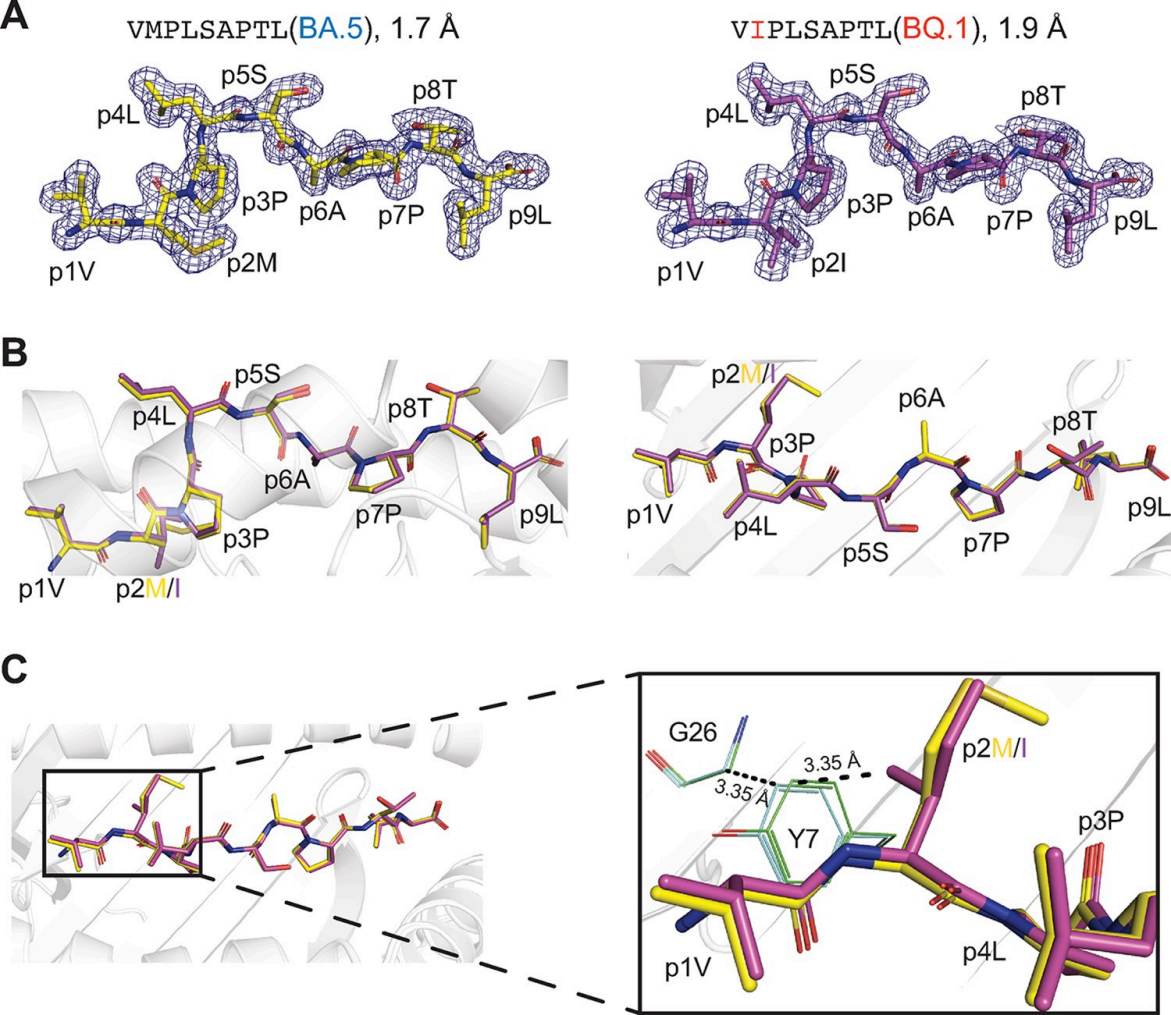
The pM2I mutation alters HLA-E B-pocket occupancy. (**A**) 2F_o_-F_c_ electron density maps of BA.5 Nsp13_232-240_ (left, yellow) and BQ.1 Nsp13_232-240_ (right, violet) in complex with HLA-E*01:03 contoured at 1.0 σ, as determined by x-ray crystallography. (**B**) Side and top views (left and right, respectively) of the superimposed crystal structures of the two Nsp13_232-240_ peptide variants. (**C**) Van der Waals distances between the CG2 atom of residue p2I in BQ.1 Nsp13_232-240_ and the HLA-E residue Y7 as well as between Y7 and G26 in the B-pocket. Inter-atom distances are indicated.

To gain deeper insights into the differential presentation of the two epitopes, we produced HLA-E*01:03 in complex with either BQ.1 Nsp13_232-240_ or BA.5 Nsp13_232-240_. Nano differential scanning fluorimetry analyses of refolded complexes showed a lower melting temperature for HLA-E/BQ.1 Nsp13_232-240_ (**S3A and S3B Fig**), reflecting poorer stability and confirming the results from the cell-based assays. Next, we used X-ray diffraction to determine the crystal structures of HLA-E*01:03/BQ.1 Nsp13_232-240_ and HLA-E*01:03/BA.5 Nsp13_232-240_ at 1.9 and 1.7 Å resolution, respectively (**Table 1 and Fig 3A**). Comparison of these high-resolution structures demonstrated that the conformations of the two epitopes were largely conserved (**Fig 3B**). Detailed examination of the HLA-E B-pocket, responsible for binding the p2 residue of the peptide, revealed that the CG2 atom branching from the side chain of the mutated isoleucine p2I in BQ.1 Nsp13_232-240_ comes into considerable proximity with the side chain of the HLA-E residue Y7. This interaction induces Y7 to tilt towards the N-terminal part of the peptide-binding cleft, rendering the van der Waals distances between p2I and Y7 as well as between Y7 and G26 unfavorable (**Fig 3C**).

**Table 1:** Data collection and refinement statistics for solved structures of HLA-E*01:03/BA.5 Nsp13_232-240_ and HLA-E*01:03/BQ.1 Nsp13_232-240_.

	HLA-E*01:03/BA.5 Nsp13_232-240_	HLA-E*01:03/BQ.1 Nsp13_232-240_
**Peptide sequence**	VMPLSAPTL	VIPLSAPTL
**PDB ID**	8RNE	8RNF
**Space group**	C 1 2 1	C 1 2 1
**Cell dimensions**		
*a* (Å)	120.5	122.2
*b* (Å)	67.6	66.5
*c* (Å)	152.2	158.4
*b* (Å)	92.8	95.3
**Resolutions (Å)**	33.35–1.71 [1.78–1.71]	41.73–1.87 [1.94–1.87]
**Total reflections**	923132 [92111]	714369 [71254]
**Unique reflections**	132141 [13193]	104375 [10235]
**Multiplicity**	7.0 [7.0]	6.8 [7.0]
**Completeness (%)**	99.8 [99.7]	99.7 [98.1]
**I/s(I)**	13.5 [1.4]	12.2 [2.6]
**R-merge**	0.08 [1.18]	0.12 [0.82]
**Complexes in au**	3	3
**Refinement**		
R_cryst_ (%)	20.1	18.0
R_free_ (%)	23.7	21.9
**Rmsd from ideal geometry**		
Bond length (Å)	0.008	0.008
Bond angles (°)	1.48	1.44
**Ramachandran plot**		
Favored (%)	98.7	98.1
Allowed (%)	1.3	1.9
Outliers (%)	0	0

### The pM2I mutation does not confer a direct inhibitory function to the Nsp13_232-240_ epitope

The ancestral sequence of the Nsp13_232-240_ epitope is efficiently presented by HLA-E but, in contrast to self-peptides, HLA-E/Nsp13_232-240_ complexes do not bind to the inhibitory receptor NKG2A. This absence of binding reduces inhibition, rendering target cells that present the viral peptide susceptible to attack by NKG2A^+^ NK cells [8]. To test whether the mutated Nsp13_232-240_ epitope may mimic self-peptides and has acquired the capacity to bind NKG2A, we loaded K562/HLA-E cells with peptide and determined the binding of recombinant NKG2A/CD94 to these cells. K562/HLA-E cells loaded with the prototypic self-peptide VMAPRTLIL (signal sequence of HLA-C; HLA-C_3-11_ [21]) strongly bound NKG2A/CD94 (**S4A Fig**), whereas both the parental BA.5 and the mutated BQ.1 Nsp13_232-240_ epitope did not show any receptor binding (**S4A Fig**). We further compared our crystal structures of the two HLA-E/Nsp13_232-240_ complexes with a previously determined structure of HLA-E in complex with the HLA-C_3-11_ self-peptide VMAPRTLIL [22] and found that the electrostatic surface potential of HLA-E/HLA-C_3-11_ was more positively charged, especially surrounding peptide positions 5 and 8 (**S4B Fig**). The self-peptide VMAPRTLIL contains an arginine at position 5 (p5R) and an isoleucine at position 8 (p8I), which enable binding to NKG2A/CD94 (**S4C Fig**) [16,18,23]. Conversely, the viral peptides VMPLSAPTL (BA.5) and VIPLSAPTL (BQ.1) share a serine at position 5 (p5S) and a threonine at position 8 (p8T), precluding direct interactions with NKG2A/CD94 (**S4C Fig**). Next, we extended our receptor binding assays with functional studies by co-culturing peptide-loaded K562/HLA-E cells with purified human NK cells and measuring CD107a surface mobilization as well as expression of the cytokines TNF-α and IFN-γ (**S4D–S4F Fig**). As expected, K562/HLA-E cells without peptide triggered robust activation of NKG2A^+^ NK cells due to absence of inhibition and loading with the VMAPRTLIL self-peptide resulted in a near-complete block of NK cell activity (**S4E and S4F Fig**). In agreement with the results of the NKG2A/CD94 binding assays, presentation of the mutated BQ.1 Nsp13_232-240_ epitope did not inhibit NKG2A^+^ NK cells and led to NK cell activity similar to the BA.5 Nsp13_232-240_ epitope (**S4E and S4F Fig**), further underscoring that the pM2I substitution did not confer a direct inhibitory function to the mutated Nsp13_232-240_ epitope.

### Impaired presentation of the mutated epitope allows for increased NK cell inhibition in mixed peptide repertoires

**Fig 4:**
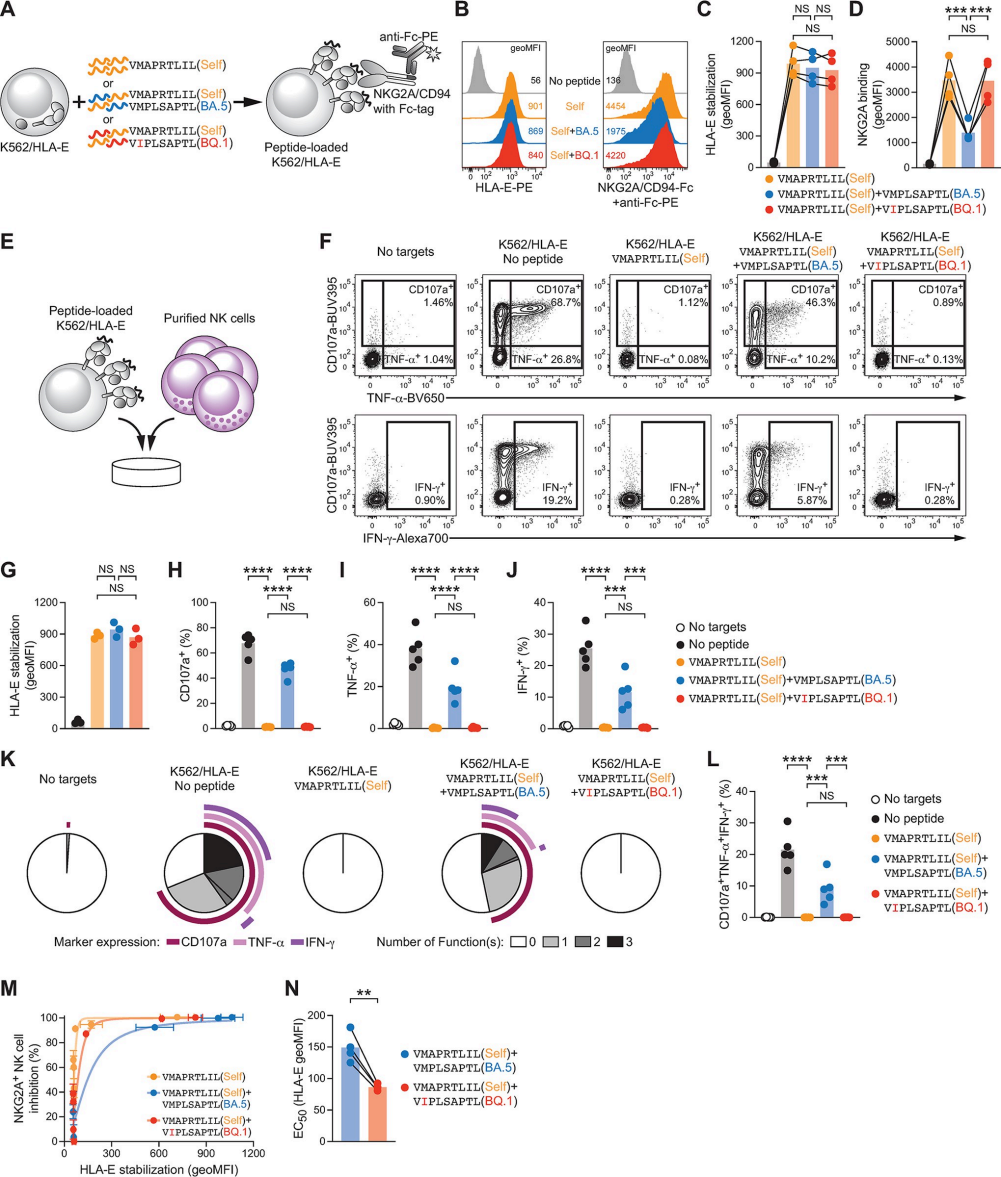
Impaired presentation of the mutated epitope from SARS-CoV-2 BQ.1 allows for increased NK cell inhibition in mixed peptide repertoires. (**A**) Schematic illustration depicting loading of K562/HLA-E cells with mixed peptide repertoires and subsequent NKG2A binding assay. Peptide concentrations within mixes were used as in **S4G Fig** and described in Methods. (**B**) Representative cell surface HLA-E levels (left) and binding of recombinant NKG2A/CD94 (right) determined by flow cytometry. Numbers denote geoMFI. (**C-D**) Summary of (C) HLA-E signals and (D) binding of recombinant NKG2A/CD94 upon loading with the indicated peptide mixes. Connected dots denote independent experiments (n = 4) and bars display mean. Repeated-measures one-way ANOVA with Šídák’s multiple comparisons test (NS p ≥ 0.05 and *** p < 0.001). (**E**) Schematic illustration depicting the NKG2A^+^ NK cell inhibition assay based on co-culture of peptide-loaded K562/HLA-E cells with purified NK cells. (**F**) Representative NKG2A^+^ NK cell activation measured by CD107a surface mobilization and expression of TNF-α as well as IFN-γ upon co-culture with K562/HLA-E cells loaded with solvent control (no peptide) or the indicated peptide mixes. Gated on viable CD3^-^ CD56^dim^ NKG2A^+^ NKG2C^-^ NK cells (gating strategy in **S4D Fig**). (**G**) Summary of HLA-E signals detected in parallel to functional assays. Dots denote independent experiments (n = 3) and bars display mean. Repeated-measures one-way ANOVA with Šídák’s multiple comparisons test (NS p ≥ 0.05). (**H-J**) Summary of NKG2A^+^ NK cell activation measured by frequency of (H) CD107a, (I) TNF-α, and (J) IFN-γ. Dots denote individual donors and bars mean (n = 5 donors in 3 independent experiments). Repeated-measures one-way ANOVA with Šídák’s multiple comparisons test (NS p ≥ 0.05, *** p < 0.001, and **** p < 0.0001). (**K**) Distributions of polyfunctional responses within NKG2A^+^ NK cells. Arcs denote mean frequency of activation marker expression and segments denote mean of cells positive for the corresponding number of markers (n = 5 donors in 3 independent experiments). (**L**) Summary of polyfunctional NKG2A^+^ NK cells co-expressing CD107a, TNF-α, and IFN-γ in the indicated conditions (n = 5 donors in 3 independent experiments). Repeated-measures one-way ANOVA with Šídák’s multiple comparisons test (NS p ≥ 0.05, *** p < 0.001, and **** p < 0.0001). (**M-N**) Peptides were mixed at a 1:9 ratio (self:viral) and K562/HLA-E cells were either treated with solvent control or loaded with 0.125, 0.5, 1.0, 100, 150, and 200 μM of peptide mixes, followed by co-culture with NK cells as in (E). Surface HLA-E levels on K562/HLA-E cells were determined in parallel to frequency of CD107a on NKG2A^+^ NK cells (see **S4I Fig**). (M) Response curve of HLA-E surface levels and normalized NKG2A^+^ NK cell inhibition. K562/HLA-E cells without peptide were set to 0% inhibition and NK cells cultured without target cells were set to 100% inhibition. Dots and error bars denote mean±SD of individual donors (n = 4 in 2 independent experiments) and lines display curve fit. (N) EC_50_ values of HLA-E levels at half-maximal inhibition. Connected dots denote individual donors and bars mean (n = 4 donors in 2 independent experiments). Paired t-test (** p < 0.01).

Our results thus far suggest that the pM2I mutation impairs the presentation of BQ.1 Nsp13_232–240_ by HLA-E without causing direct inhibition of NK cells in a peptide-intrinsic manner. Although viruses including SARS-CoV-2 can commandeer the translation machinery of infected cells [24], peptides derived from host proteins remain a considerable part of the presented repertoire, providing an opportunity for competition and peptide antagonism within a mixed peptide repertoire [25]. To model the impact of the BQ.1 Nsp13_232-240_ epitope and its impaired presentation profile in the context of a mixed peptide repertoire, we loaded K562/HLA-E cells either with self-peptide alone or with mixes of self-peptide and viral Nsp13_232-240_ variants (**Fig 4A**). We then titrated different concentrations of self-peptide while maintaining Nsp13_232-240_ concentrations constant, generating conditions with similar HLA-E surface level, allowing us to study the impact of the peptide composition rather than the overall magnitude of HLA-E stabilized on the surface (**Figs 4B, 4C and S4G**). Receptor binding assays revealed that at a comparable total surface HLA-E level, mixes of self-peptide with BQ.1 Nsp13_232-240_ allowed for a more pronounced binding of recombinant NKG2A/CD94 than mixes of self-peptide with BA.5 Nsp13_232-240_ (**Figs 4B and 4D**). To ascertain that the selected HLA-E level does not affect these data, we repeated the receptor binding assays at different concentrations of the mixed peptide repertoire and obtained concordant NKG2A binding patterns (**S4H Fig**).

To directly probe whether the presentation of a mix of self-peptide and BQ.1 Nsp13_232-240_ recovers inhibition of NKG2A^+^ NK cells, we loaded K562/HLA-E cells with peptide mixes that result in identical HLA-E levels, followed by co-culture with purified human NK cells (**Fig 4E**). Compared to mixes consisting of self-peptide and BA.5 Nsp13_232-240_, presence of BQ.1 Nsp13_232-240_ resulted in less pronounced activation of NKG2A^+^ NK cells in all measured parameters including polyfunctionality (**Fig 4F–4L**), likely reflecting increased inhibition mediated by the self-peptide within the mixed peptide repertoire.

Finally, to evaluate mixed peptide repertoires over a broad range of HLA-E levels, we mixed self-peptide and Nsp13_232-240_ variants at a constant ratio and loaded K562/HLA-E cells with varying concentrations of these mixes. Thereafter, we determined HLA-E stabilization on the surface of the target cells and, in parallel, monitored NK cell responses the co-cultures with target cells (**S4I Fig**). Assessing NK cell inhibition as a dose-response of the corresponding HLA-E stabilization for each condition exposed that self-peptide alone efficiently inhibited NK cells already at concentrations that induced only a minor stabilization of HLA-E on the target cell surface (**Fig 4M**). Conversely, peptide mixes containing BA.5 Nsp13_232-240_ required higher HLA-E levels to achieve similar inhibition, while mixes with BQ.1 Nsp13_232-240_ showed an intermediate efficiency, requiring significantly less HLA-E stabilization for half-maximal inhibition of NKG2A^+^ NK cell activity (**Fig 4N**).

Together, these findings suggest that BA.5 Nsp13_232-240_ and BQ.1 Nsp13_232-240_ differentially impact NK cell responses within mixed peptide repertoires, with the pM2I mutation facilitating enhanced simultaneous presentation of self-peptide and correlating with elevated NKG2A binding as well as increased NK cell inhibition.

### The pM2I mutation associates with enhanced estimated viral fitness and is transmitted to descendant sub-lineages

**Fig 5:**
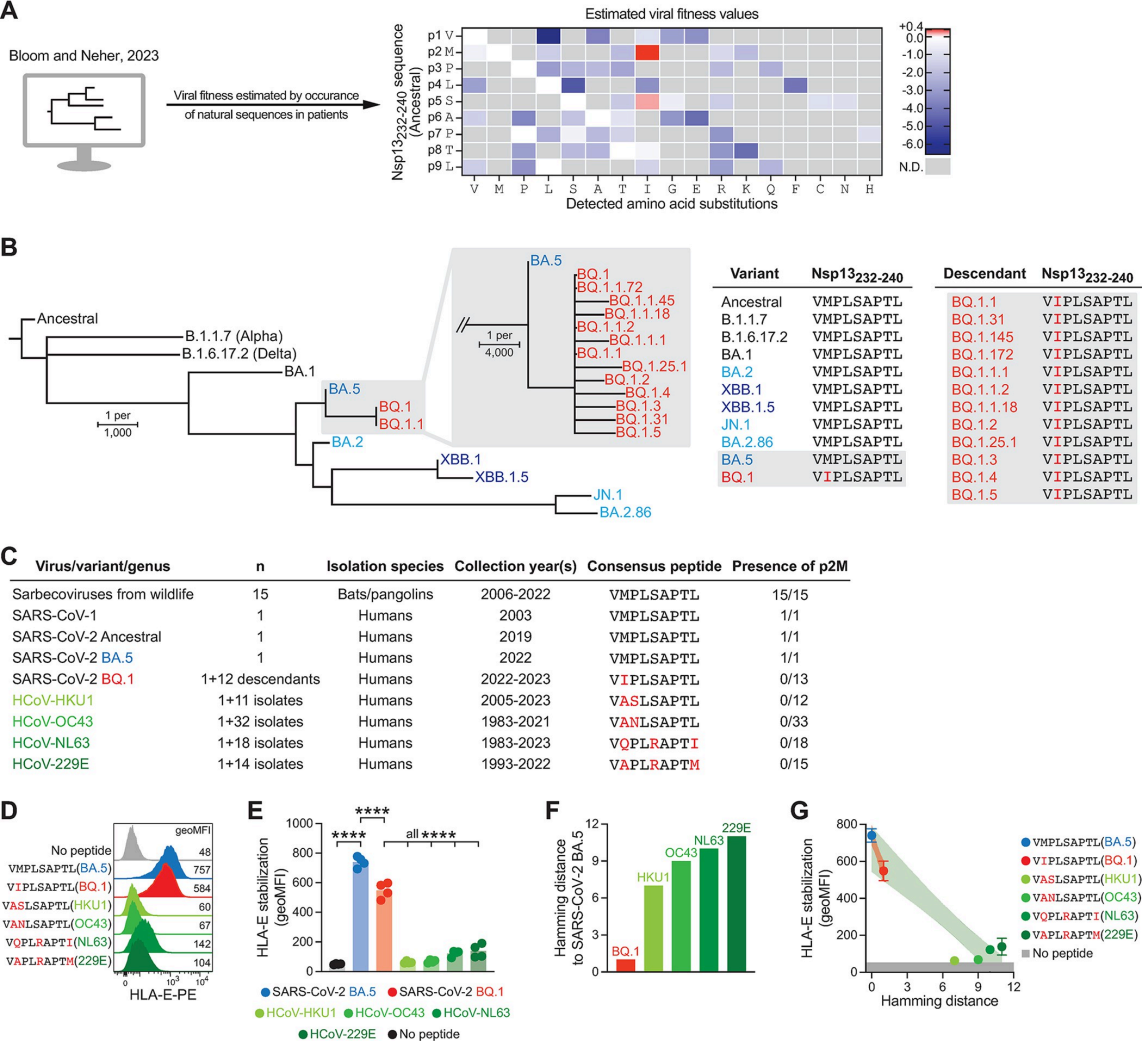
The pM2I mutation is associated with enhanced viral fitness and resembles peptides found in common cold-causing human coronaviruses. (**A**) Viral fitness estimated by comparing the observed occurrence of each mutation with its anticipated frequency based on the neutral mutation rate of SARS-CoV-2. Data is based on 6.5 million publicly available SARS-CoV-2 genomes [26, 27]. Every observed amino acid substitution within the Nsp13_232-240_ epitope except Stop codons are displayed. Grey tiles indicate not detected (N.D.) amino acids. (**B**) Phylogenic tree illustrating genomic divergence between SARS-CoV-2 variants as determined by Clustalω. Tabular inlets display the sequence of the corresponding Nsp13_232-240_ epitopes in (left) variants and (right) descendants of BQ.1. (**C**) Tabular summary of peptides related to the Nsp13_232-240_ epitope as found in sarbecoviruses from wildlife, SARS-CoV-1, SARS-CoV-2 variants, and common cold-causing human coronaviruses. Amino acid alterations are highlighted in red compared to SARS-CoV-2 BA.5 Nsp13_232-240_ and the presence of methionine in position two of the peptide (p2M) is indicated in the last column. See S1 Table for a complete list. (**D**) Representative cell surface HLA-E levels determined by flow cytometry following loading with viral peptides. (**E**) Summary of HLA-E signals upon loading of the indicated peptides. Dots denote individual experiments and bars mean (n = 4 independent experiments). Repeated-measures one-way ANOVA with Šídák’s multiple comparisons test (**** p < 0.0001). (**F**) Hamming distance of the peptide-encoding genomic sequence relative to SARS-CoV-2 BA.5. See S5 Fig. (**E**) Correlation between Hamming distance as in (F) and HLA-E levels as in (E). Dots and error bars denote mean±SD (n = 4 independent experiments). Colored areas indicate 95% confidence bands of linear regression between BA.5 and BQ.1 in red (best fit slope±SE –192±32) as well as BA.5 and HCoV in green (best fit slope±SE –59±6.9).

Considering that the pM2I mutation diminishes presentation of Nsp13_232-240_ by HLA-E and allows for more NK cell inhibition by self-peptide, we asked whether its presence is advantageous for SARS-CoV-2. To address this question, we employed a recently described *in silico* approach serving as a proxy for estimating viral fitness by analyzing naturally occurring viral sequences [26]. This computational estimation is based on millions of publicly available genomes isolated from patients [27], providing sufficient data to define the neutral mutation rate of SARS-CoV-2 and therefore allowing the mathematical comparison of the anticipated frequency of each mutation with its observed occurrence in virus genomes isolated from patients [26]. Analyses of all substitutions detected within the Nsp13_232-240_ epitope in all available SARS-CoV-2 genomes showed that most mutations exhibited deleterious effects, resulting in reduced estimated fitness. In contrast, the pM2I mutation associated with enhanced fitness values (**Fig 5A**), indicating a distinctively advantageous role not only in the BQ.1 sub-variant, but across the entire spectrum of SARS-CoV-2 genomes. This notion is further substantiated by the observation that all of 12 tested descendent sub-lineages of BQ.1 encode for the VIPLSAPTL sequence and hence have not reversed to the parental VMPLSAPTL epitope (**Fig 5B**).

### The pM2I mutation of SARS-CoV-2 Nsp13_232-240_ resembles peptides found in common cold-causing human coronaviruses

Since SARS-CoV-2 has recently emerged and is rapidly adapting to humans as its host, we next contextualized our findings by comparing the Nsp13_232-240_ epitope of SARS-CoV-2 with peptides found in related viruses (**S1 Table**). Sarbecoviruses found in wildlife such as bats and pangolins are closely related to SARS-CoV-2 [12]. Surveying genomes of wildlife sarbecoviruses sampled over a period of 17 years, we found that 14 out of 15 viruses encode for the VMPLSAPTL epitope that is identical to the ancestral SARS-CoV-2 epitope. Furthermore, all 15 wildlife viruses share a methionine in position 2 of the peptide sequence (p2M; **Fig 5C**). SARS-CoV-1, which caused an outbreak in humans between 2002 and 2004, is another virus closely related to SARS-CoV-2 and likewise encoded the VMPLSAPTL sequence (**Fig 5C**). Notably, the four common cold-causing HCoV NL63, OC43, 229E, and HKU1, which have been endemic in humans for decades, contain similar peptide sequences. Strikingly, however, these four HCoV do not encode for p2M, but rather show divergent amino acids at this key anchor position (**Fig 5C**), paralleling the pM2I mutation found in the BQ.1 variant of SARS-CoV-2. To explore dynamics within HCoV, we examined the sequences of 75 isolates collected from patients during a period of 40 years. This analysis confirmed a fully conserved absence of p2M that was stable over time without any turnover in these endemic viruses (**Fig 5C and** **S1 Table**).

Upon assessing the HLA-E presentation capacity of viral peptides in cellular presentation assays, we found that Nsp13_232-240_ from SARS-CoV-2 BA.5 - which is shared with ancestral SARS-CoV-2, SARS-CoV-1, and sarbecoviruses from wildlife—displayed the most pronounced HLA-E binding capacity (**Fig 5D and 5E**), likely due to the presence of p2M. In stark contrast, the four HCoV peptides that lack p2M bound poorly to HLA-E and the SARS-CoV-2 BQ.1 Nsp13_232-240_ epitope carrying the pM2I mutation showed an intermediate presentation profile, positioned between the BA.5 epitope and the HCoV peptides (**Fig 5D and 5E**).

To obtain a quantitative understanding of the concordance among these viral peptides, we calculated the Hamming distance [28,29] of the peptide-encoding genomic regions with SARS-CoV-2 BA.5 as a reference (**Figs 5F and** S5). Next, we set the experimentally observed HLA-E presentation in relation to their Hamming distance (**Fig 5G**). This comparison uncovered a sharp decline in HLA-E presentation between SARS-CoV-2 BA.5 and the BQ.1 variant that was more pronounced than the decrease observed between SARS-CoV-2 BA.5 and HCoV (best fit slope±SE –192±32 for BQ.1 and –59±6.9 for HCoV; linear regression), implying a disproportionately effective reduction in peptide presentation by the pM2I substitution.

Collectively, these data indicate impaired peptide presentation as adaption of SARS-CoV-2 to evade NK cell-mediated immunosurveillance by restoring NKG2A-dependent inhibition of NK cells.

## Discussion

In this study, we report that a single amino acid substitution in an HLA-E-restricted epitope diminishes its presentation and correlates with recovered NK cell inhibition.

The emergence of the SARS-CoV-2 Omicron variant unveiled a striking degree of antigenic novelty [30–32], representing a major adaptation to host immunity and enabling immune evasion. While viral fitness is governed by multiple factors beyond immune evasion, such as infectivity and replicative capacity, the fitness of sub-lineages is dependent on subtle changes in antigenicity. Such dependence on subtle differences may become especially pronounced when the virus encounters a high degree of immunity within its host population, for example generated by vaccination or prior infection. Therefore, it is plausible that diminished presentation of a peptide that renders infected cells susceptible to NK cell attack, represents an adaptation that confers a subtle yet impactful competitive advantage to the virus and contributes to viral spread and dominance over other circulating sub-lineages.

The primary function of HLA-E has been ascribed to presenting self-peptides to NK cells [10]. Since presented self-peptides are mainly leader sequences of other HLA class I molecules such as HLA-A, -B-, or -C [14,21], HLA-E/peptide complexes act as a surrogate for monitoring adequate HLA class I expression in healthy cells. Complexes of HLA-E and self-peptide serve as ligands for the inhibitory receptor NKG2A, ensuring that healthy cells presenting appropriate peptide ligands are protected from NK cell attack [21]. In addition to self-peptides, HLA-E can also present pathogen-derived peptides [10], which may compete with endogenous self-peptides for presentation if they exhibit high affinity towards HLA-E. In cases where pathogen-derived peptides are prominently presented but fail to bind NKG2A, they can antagonize inhibition and thereby facilitate activation of NK cells [8,33,34]. The phenomenon of peptide antagonism [25], in which changes in peptide content within a mixed peptide repertoire result in reduced inhibition and, in turn, promote NK cell activation, represents an indirect activation mechanism of NK cells. As such, peptide antagonism parallels missing-self recognition, where NK cells respond to the absence of self-ligands and are robustly activated due to loss-of-inhibition [35].

NK cells have been implicated in shaping the evolution of viruses such as HIV, where viral epitopes are selected for enhanced binding to inhibitory NK cell receptors, thereby achieving evasion from NK cell activity through direct inhibition [36,37]. In contrast to the findings in HIV, the pM2I mutation in the Nsp13_232-240_ epitope of SARS-CoV-2 has not acquired features that result in direct inhibition of NK cells. Instead, the diminished presentation of the mutated epitope decreases NK cell activity indirectly, by allowing for heightened concomitant presentation of inhibitory self-peptides. Thus, through a single amino acid substitution, competition for presentation with endogenous self-peptides is curtailed, permitting a recovery of inhibition of NK cell activity.

Evolutionary selection of peptide sequences that maintain presentation by HLA-E and acquire the capacity to effectively bind NKG2A represents a potential alternative to evolution towards impaired presentation. Such pathogen-derived peptides mimicking self-peptides and functionally suppressing NK cell activity are found for instance in human cytomegalovirus (CMV) [38]. However, these peptides concurrently trigger the HLA-E/peptide-specific activating receptor NKG2C [39], driving the activation of another NK cell sub-population that is capable of controlling CMV infection [40]. Therefore, the net benefit for the pathogen is likely determined by a fine balance between antagonizing inhibition and triggering activation. Molecular evolution yielding in diminished presentation bypasses both recognition modes simultaneously, achieving immune escape from NKG2A^+^ as well as NKG2C^+^ NK cells. Moreover, paucity of peptide presentation excludes HLA-E-restricted T cell responses [13], enabling comprehensive evasion from cytotoxic immune cells.

Embedding the discovered molecular evolution of SARS-CoV-2 Nsp13_232-240_ into a broader context of related coronaviruses revealed that sarbecoviruses from wildlife and SARS-CoV-1, which briefly circulated among humans in a restricted outbreak, share the ancestral SARS-CoV-2 epitope. In contrast, common cold-causing HCoV contain sequences related to the SARS-CoV-2 epitope, albeit with strikingly poorer presentation by HLA-E. Interestingly, none of the HCoV peptides harbor the canonical methionine at the key anchor position 2, resembling the pM2I mutation found in the BQ.1 sub-variant of SARS-CoV-2. Furthermore, HCoV peptides contain additional divergent amino acids at position 3 (OC43 and HKU1) or at positions 5 and 9 (229E and NL63). Since HCoV have been endemic in humans for at least several decades, it is tempting to speculate that protracted periods of circulation have favored amino acids at anchor positions that prevent presentation by HLA-E, potentially due to immune pressure. The fact that an analogous mutation at a key anchor position of SARS-CoV-2 Nsp13_232-240_ emerged within a short timeframe of circulation in humans underscores its remarkably rapid adaptation.

Our study provides insights into the presentation efficiency, structural features, and functional consequences of SARS-CoV-2-encoded HLA-E-binding peptides. The lack of evidence for differential *in vivo* control of SARS-CoV-2 variants by NK cells can be regarded as a limitation. While studying SARS-CoV-2 *in vivo* is generally challenging, translating HLA-E-restricted responses into animal models poses a particular challenge since HLA-E is specific to humans, and its homologues Qa-1b in mice or Mamu-E in rhesus macaques display distinct peptide binding profiles [41]. Likewise, NK cell responses against infected cell lines *in vitro* are complex settings with multiple signals acting simultaneously, including potential allogeneic and cancer cell-driven NK cell responses, which may obscure differences between virus strains. The virus fitness data presented in this study are computationally obtained estimations and have not been tested head-to-head in a controlled laboratory setting. However, since the fitness estimations are based on genomic sequences isolated from patients, the occurrence of mutations in genomic sequence databases relates to viruses that have been sufficiently fit to infect humans, albeit indirectly [26]. Nonetheless, future studies employing novel animal models and genetically engineered viruses that can overcome the challenges noted above will aid in fully dissecting these aspects.

Collectively, our findings demonstrate that a mutation in the Nsp13_232-240_ epitope diminishes its presentation by HLA-E and correlates with recovered inhibition of NK cells. These observations underscore the rapid adaptation of SARS-CoV-2 to immune responses and imply that NK cell recognition mediates evolutionary pressure onto the virus. We propose that continuous monitoring of emerging variants may provide deeper insights into viral adaptation to innate immunity and is likely to expose additional critical targets of immune pressure.

## Materials and methods

### Ethics statement

Buffy coats from healthy volunteer blood donors, who gave written consent, were obtained from the Department of Clinical Immunology and Transfusion Medicine, Karolinska Institutet as approved by the Ethical Review Board Stockholm (DNR 2020–05289).

### SARS-CoV-2 variant data

Publicly available data of SARS-CoV-2 variant frequencies were downloaded from ECDC (https://www.ecdc.europa.eu/en/publications-data/data-virus-variants-covid-19-eueea) on 2023-06-02. The absolute numbers reported from Belgium, France, Germany, Norway, Portugal, Spain, and Sweden were combined and frequencies were calculated. For simplicity, BQ.1 and its descendant BQ.1.1 were combined. Data reported from Sweden were correct according to the Public Health Agency of Sweden (Folkhälsomyndigheten, weekly report 39 in 2022). Data on patients with COVID-19 being treated on intensive care units in Sweden were downloaded from the data portal of the Swedish Intensive Care Registry (Svenska Intensivvårdsregistret) on 2024-07-08 (https://portal.icuregswe.org/siri/sv/report/corona_covid-dagligen).

### Cells and cell lines

PBMC were isolated from buffy coats with standard density gradient centrifugation, cryopreserved in FBS containing 10% (V/V) DMSO, and stored in the vapor phase of liquid nitrogen as described previously [42]. K562 cells expressing HLA-E*01:03 (K562/HLA-E) were generated by E. Weiss, Ludwig Maximilian University [43]. K562/HLA-E cells were maintained in complete RPMI (RPMI-1640 supplemented with 2 mM glutamine, 10% [v/v] FBS, 100 U/mL penicillin, and 100 μg/mL streptomycin; all Gibco) in the presence of 1 mg/mL G418 (Gibco). Caco-2 cells were a kind gift from Soham Gupta, Karolinska Institute, and maintained in complete DMEM (DMEM supplemented with 2 mM glutamine, 10% [v/v] FBS, 100 U/mL penicillin, and 100 μg/mL streptomycin; all Gibco). A549 cells expressing angiotensin-converting enzyme 2 (ACE2; A549-hACE2) were generated by Alba Corman, Bartlomiej Porebski, and Oscar Fernandez-Capetillo, Karolinska Institute. A549-hACE2 cells were maintained in complete MEM (MEM supplemented with 2 mM glutamine, 10% [v/v] FBS, 100 U/mL penicillin, and 100 μg/mL streptomycin; all Gibco). Cell lines were routinely tested for presence of Mycoplasma (Mycoplasmacheck, Eurofins Genomics).

### Peptides

Lyophilized synthetic peptides with purity ≥95% (Peptides&Elephants, Genscript, or JPT; **S3 Table**) were reconstituted in sterile water at 12 mM. Lysine-substituted peptides were obtained at crude purity (Peptides&Elephants).

### Cellular peptide presentation assays

Loading of K562/HLA-E cells was performed as described previously [8,39]. In brief, K562/HLA-E cells were cultured at 2x10^6 cells per mL in serum-free Opti-MEM (Gibco) supplemented with 100 U/mL penicillin, and 100 μg/mL streptomycin (both Gibco). Synthetic peptides were added at the indicated final concentration and the cells were incubated in U-bottom well plates at 37°C over-night (14–18 h). As solvent control, the appropriate volume of sterile water was added (referred to as no peptide). Peptide-loaded cells were either washed with PBS and stained for flow cytometric analyses of HLA-E surface levels or washed with complete RPMI for use in co-cultures.

### Cellular HLA-E/peptide stability assays

Assessment of HLA-E/peptide complex stability and turnover was performed by pulsing K562/HLA-E cells with 300 μM peptide in Opti-MEM at 37°C over-night (as above), followed by removal of the peptide-containing Opti-MEM and one wash with Opti-MEM without peptides. Subsequently, the cells were cultured in Opti-MEM without peptides at 37°C. After the indicated times, cells were washed with PBS and stained for flow cytometric analyses of HLA-E surface levels.

### *In vitro* infection

Caco-2 and A549-hACE2 cells were inoculated with SARS-CoV-2 Ancestral (isolate SARS-CoV-2/human/SWE/01/2020; Genbank accession MT093571.1) or SARS-CoV-2 BQ.1 (reference strain hCoV-19/France/HDF-IPP49210/2022; EVAg identifier 017V-04968), as described previously [8]. In brief, cells were seeded in 48 well plates under biosafety level 2 conditions. After 24 to 72 h, the cells were transferred to a biosafety level 3 facility, washed with PBS, and inoculated with virus in serum-free media (DMEM for Caco-2 and MEM for A549-hACE2). After 1 h, virus-containing media was removed, the cells were washed with complete DMEM or complete MEM, and thereafter maintained in complete DMEM or complete MEM. At the indicated time points post infection, the cells were washed with PBS, detached with Trypsin-EDTA solution (Merck), washed in complete DMEM or complete MEM, stained for flow cytometric analyses for 20 min at 4°C, and fixed with Cytofix/Cytoperm (BD Biosciences) containing 4.2% (w/w) formaldehyde.

### Production, purification, and crystallization of HLA-E/peptide complexes

HLA-E*01:03 heavy chain and human β_2_m (hβ_2_m) were expressed individually as inclusion bodies using BL21(DE3) E. coli as previously described [44–46]. Inclusion bodies were solubilized in 8 M Urea, 100 mM Tris HCl pH 8, and 2 mM EDTA. Refolding of HLA-E*01:03 with hβ_2_m and either BA.5 Nsp13_232-240_ or BQ.1 Nsp13_232-240_ was initiated by resuspending 10 mg peptide in 5 mL refolding buffer (100 mM Tris, 400 mM L-arginine, 2 mM EDTA, 0.5 mM glutathione disulfate, 5 mM glutathione and 0.5 mM PMSF), and added dropwise to 1 L buffer on a magnetic stirrer at 4°C. A total of 24 mg hβ_2_m was added to the refolding mixture after peptide injection. After 30 minutes, 10 mg of HLA-E*01:03 heavy chain was injected three consecutive times with three-hour intervals. After 24 hours, aggregates were removed using a rapid-filtermax 0.22 μm vacuum filter (ThermoFisher) and the refolded HLA-E*01:03/peptide complexes were concentrated using first a peristaltic pump (Vivaflow 200, 30K MWCO, Sartorius) and thereafter centrifugal filtration with a 30K filter (Vivaspin). Finally, HLA-E*01:03/peptide complexes were purified using size exclusion chromatography with a Superdex 200 Prep Grade on an Äkta Purifier (Cytiva), followed by SDS-PAGE validation. Fractions containing HLA-E*01:03/peptide complexes were pooled, concentrated, flash frozen in liquid nitrogen, and stored at -80°C. Crystals of the HLA-E*01:03/peptide complexes were obtained and optimized using sitting drop vapor diffusion and micro seeding methods at 20°C. A total of 100 nL complex (10 mg/mL) were mixed with 100 nL reservoir buffer in crystallization wells and equilibrated by sitting drop vapor-diffusion at 20°C. Commercial sparse matrix grid screens were used to identify optimal crystallization conditions, around which ammonium sulphate fine gradient and additive screens were subsequently setup. Specifically, 0.12 μL of 10 mg/mL complex were mixed with 0.15 μL of reservoir solution and 0.03 μL seeds from unoptimized HLA-E*01:03/peptide crystals. Crystals of HLA-E*01:03/BA.5 Nsp13_232-240_ grew in 0.15 M potassium bromide, 20% (w/V) PEG 2000 MME, while crystals of HLA-E*01:03/BQ.1 Nsp13_232-240_ were obtained in 0.15 M DL malic acid and 20% (w/V) PEG 3350. Crystals were cryopreserved in 25% (w/V) glycerol.

### Data collection, processing, and structure determination

Diffraction data collection was performed at European Synchrotron Radiation Facility for both HLA-E*01:03/peptide complexes. Data collection statistics are presented in Table 1. Crystal structures were determined by molecular replacement using Phaser MR [47] and the HLA-E*01:01/VMAPRTVLL structure (PDB ID 1MHE) [16] stripped of peptide, hydrogens, and water molecules as the search model. Structures were refined using Refmac5 [48] and Phenix [49] with inspection and correction in COOT [50]. Randomly selected 5% of reflections were used for monitoring refinement by free R cross-validation [51]. Refinement statistics are provided in Table 1. The final structural models and figures were generated using the PyMOL Molecular Graphics System, 2.5.0a Open-Source (Schrödinger, LLC) and the previously solved structure of HLA-E*01:03/HLA-C_3-11_ (PDB ID 5W1V) [22] was used as reference for the surface electrostatic potential.

### Molecular modeling of CD94/NKG2A and HLA-E*01:03/peptide complexes

To assess the role of residues in HLA-E*01:03 and the Nsp13_232-240_ peptides in engaging the CD94/NKG2A heterodimer, the crystal structure of HLA-E*01:03/BA.5 Nsp13_232-240_ was superposed on the previously determined crystal structure of the CD94/NKG2A/HLA-E*01:01 complex (PDB ID 3CII) [23], with the presented peptide mutated to HLA-C_3-11_ VMAPRTLIL using COOT [52]. The superposition was based on the Ca atoms of heavy chain residues 3–176, assuming that CD94/NKG2A would interact with HLA-E/Nsp13_232-240_ complexes similarly to other reported HLA-E/peptide complexes.

### Nano-differential scanning fluorimetry (nano-DSF)

Thermal unfolding experiments were performed using nano-DSF as previously described [53]. The protein intrinsic fluorescence during the thermal ramp was followed at 330 and 350 nm with a Prometheus NT.48 instrument from NanoTemper Technologies with an excitation wavelength of 280 nm. Capillaries were loaded with 10 μL of protein at a concentration of 1 mg/mL in 20 mM Tris HCl, pH 8.0, and 150 mM NaCl. Temperature ramp measurements were recorded from 20 to 95°C (with a temperature slope of 60°C/hour). Three independent measurements were performed for each HLA-E*01:03/peptide complex. The fluorescence intensity ratio was recorded, and its first derivative was calculated using GraphPad Prism.

### Recombinant NKG2A binding assay

For assessing receptor binding, peptide-loaded K562/HLA-E cells were incubated with 25 μg/mL recombinant NKG2A/CD94 heterodimers carrying a human IgG1 Fc-tag at the C-terminus (Acro Biosystems) for 45 min on ice, followed by detection with anti-human IgG Fc-PE secondary antibody (**S2 Table**). To test NKG2A binding to K562/HLA-E cells presenting mixed peptide repertoires, conditions were selected for similar HLA-E levels as displayed in **S4G Fig** (comparison in Fig 4B–4D are 300 μM VMAPRTLIL vs. 100 μM VMPLSAPTL+100 μM VMAPRTLIL vs. 100 μM VIPLSAPTL+200 μM VMAPRTLIL and in **S4 Fig** 400 μM VMAPRTLIL vs. 100 μM VMPLSAPTL+200 μM VMAPRTLIL vs. 100 μM VIPLSAPTL+250 μM VMAPRTLIL).

### Functional NK cell inhibition assay

Assays to determine whether viral peptides or mixes of viral and self-peptides presented on HLA-E inhibit functional responses of NKG2A-expressing NK cells were performed as described previously [8,42]. In brief, NK cells were enriched from PBMC by negative selection using the NK cell isolation kit (Miltenyi). NK cells were maintained over-night (15–18 h) in complete RPMI supplemented with 1 ng/mL recombinant human IL-15 (RnD). The next morning, media was replaced with complete RPMI without cytokine and 1x10^5 NK cells were co-cultured with 1x10^5 peptide-loaded K562/HLA-E cells in V-bottom 96 well plates in the presence of anti-human CD107a antibodies (**S2 Table**). To assess NK cell responses to single peptides, K562/HLA-E cells were loaded with 100 μM peptide, and for mixed peptide repertoires, K562/HLA-E cells were either loaded with concentrations selected for similar HLA-E levels (Fig 4E–4L) or with mixes of constant ratios over a broad range of concentrations (Fig 4M and 4N; comparisons in Fig 4E–4L: 250 μM VMAPRTLIL vs. 200 μM VMPLSAPTL+0.25 μM VMAPRTLIL vs. 200 μM VMPLSAPTL+50 μM VMAPRTLIL; comparisons in Fig 4M and 4N: mixes with self:virus ratio of 1:9 and concentrations of 0.125, 0.5, 1.0, 100, 150, and 200 μM). Peptides were re-added into the stimulation to achieve the same final concentration as in the loading. After 1 h, GolgiStop and GolgiPlug (both BD Biosciences) were added, followed by 3 more h of co-culture. Subsequently, cells were washed with PBS and stained for flow cytometric analyses. NKG2A^+^ NK cells were identified as single viable CD3^-^ CD16^+^ CD56^dim^ NKG2C^-^ NKG2A^+^ lymphocytes (gating strategy in **S4D Fig**).

### Flow cytometry

Flow cytometric stainings and analyses were performed following established guidelines [54]. In brief, cell suspensions were incubated with combinations of fluorochrome-conjugated antibodies (**S2 Table**) at optimized concentrations in PBS for 20 min at RT. Viable cells were identified with LIVE/DEAD Fixable Aqua Dead Cell Stain Kit (ThermoFisher). All samples were fixed before acquisition using BD Cytofix/Cytoperm (BD Biosciences) according to the manufacturer’s instructions. For intracellular detection of IFN-γ and TNF-α, surface-stained cells were fixed and permeabilized with BD Cytofix/Cytoperm (BD Biosciences), followed by intracellular staining in BD Perm/Wash (BD Biosciences) overnight (15–18 h) at 4°C. Samples were acquired on an LSR Fortessa flow cytometer and analyzed with FlowJo v10.9.0 (both BD Biosciences). Polyfunctional NK cell responses were analyzed and displayed using SPICE [55].

### Peptide binding prediction

Prediction of peptide binding to HLA-E was performed as previously described [8]. In brief, NetMHC4.0 (https://services.healthtech.dtu.dk/services/NetMHC-4.0/) [56,57] was used to obtain binding affinities of nonamer sequences to HLA-E*01:01. Binding scores were calculated by dividing 100 by the predicted affinity in nM.

### Phylogenic analyses

To examine phylogenic relationships, representative genomic sequences of SARS-CoV-2 variants were obtained from public databases (Genbank and GISAID, **S1 Table**). Multiple sequence alignments were performed at EMBL-EBI [58] using Clustalω [59] (RRID: SCR_001591; https://www.ebi.ac.uk/Tools/msa/clustalo/) and phylograms were generated with iTOL [60] (RRID: SCR018139; https://itol.embl.de/).

### Estimation of viral fitness

Computational estimation of viral fitness per site and substitution have been recently described [26]. In brief, the anticipated frequency of each mutation based on the neutral mutation rate of SARS-CoV-2 was compared with its observed occurrence in 6.5 million sequenced SARS-CoV-2 isolates from patients. Estimated fitness values were calculated as the natural logarithm of the ratio of actual to expected counts after adding a pseudo-count of -0.5 to each. Thus, ancestral residues have a value of zero, and all observed amino acid substitutions have either negative values indicating reduced fitness or positive values suggestive of enhanced fitness. The values for mutations within the Nsp13_232-240_ epitope were obtained from https://jbloomlab.github.io/SARS2-mut-fitness/public_2023-05-11/index.html on 2023-06-10.

### Comparisons across various viruses

To compare the similarity of peptides encoded by viruses related to SARS-CoV-2, genomes of sarbecoviruses from wildlife, SARS-CoV-1, SARS-CoV-2 variants, and common cold-causing human coronaviruses (HCoV) were obtained from public databases (Genbank and GISAID, **S1 Table**). Pairwise Hamming distances were calculated as described previously [28,29] with the peptide-encoding genomic sequence of SARS-CoV-2 BA.5 as reference (S5 **Fig**).

### Quantification & statistics

Statistical parameters (sample size, number of performed experiments, employed statistical tests, and statistical significance) are reported in the figures and figure legends, with p ≥ 0.05 considered not significant (NS) and * p <  0.05, ** p <  0.01, *** p < 0.001, and **** p < 0.0001. In general, two groups of paired samples were analyzed with paired t-test and three or more groups of paired samples were analyzed with repeated-measures one-way ANOVA followed by Šídák’s multiple comparisons test. Paired samples with two variables (e.g., different peptides and different concentrations) were analyzed with repeated-measures two-way ANOVA with Šídák’s multiple comparisons test. If not stated otherwise, statistical analyses were performed in Prism 10 (GraphPad Software) with a confidence level of 0.95. EC_50_ and half-life values were determined by Prism10 (GraphPad Software) using non-linear regression models (agonist response with variable parameters, agonist normalized response with variable slope, and one-phase-exponential decay, respectively).

## Supporting information

S1 Fig SARS-CoV-2 Omicron BQ.1 harbors a single nucleotide substitution in the sequence encoding the Nsp13_232-240_ epitope. Genomic sequences of ancestral SARS-CoV-2 and Omicron sub-lineages around the Nsp13_232-240_-encoding sequence.
(PNG)

S2 Fig Anchor positions within BA.5 Nsp13_232-240_ and *in vitro* infection experiments. (**A, B**) Lysine scanning along the BA.5 Nsp13_232-240_ peptide sequence. (A) Predicted HLA-E binding scores and (B) measured surface HLA-E levels upon loading of K562/HLA-E cells with indicated peptides. Dots denote individual experiments and bars mean (n = 3 independent experiments). (**C**) Summary of surface HLA-E levels over time on Caco-2 cells following infection with MOI = 5. Dots denote means and error bars SEM (n = 4 infections in n = 2 independent experiments). Two-way ANOVA with Šídák’s multiple comparisons test (* p < 0.05). (**D**) Summary of surface HLA-E levels over time on A549-hACE2 cells following infection with MOI = 5. Dots denote means and error bars SD (n = 3 infections in n = 1 independent experiment). Two-way ANOVA with Šídák’s multiple comparisons test (NS p ≥ 0.05 and * p < 0.05).
(PNG)

S3 Fig Melting temperatures of HLA-E/peptide complexes. (**A**, **B**) Nano differential scanning fluorimetry analyses of (A) HLA-E*01:03/BA.5 Nsp13_232-240_ and (B) HLA-E*01:03/BQ.1 Nsp13_232-240_. Arrows denote inflection points, from which melting temperatures were derived. Representative curves of triplicate experiments are displayed.
(PNG)

S4 Fig The pM2I mutation does not confer a direct inhibitory function to the Nsp13_232-240_ epitope. (**A**) Representative binding of recombinant NKG2A/CD94 as determined by flow cytometry upon loading of K562/HLA-E with 300 μM of indicated peptides. Numbers denote geoMFI. (**B**) Top view of surfaces of HLA-E/peptide complexes. Red and blue indicate negative and positive electrostatic charges, respectively. Peptide positions 5 and 8 as well as HLA-E positions 62, 65, and 152 are indicated for orientation. (**C**) Superimposition of the HLA-E*01:03/BA.5 Nsp13_232-240_ crystal structure on a previously determined structure of an HLA-E*01:01/HLA-C_3-11_ complex engaging CD94/NKG2A (PDB ID 3CII; Kaiser *et al*.). HLA-C_3-11_ and SARS-CoV-2 BA.5 Nsp13_232-240_ are colored in orange and yellow, respectively. HLA-C_3-11_ arginine and isoleucine in position 5 and 8, respectively, as well as residue Q112 of CD94 are indicated for clarity. (**D**) Gating strategy for NKG2A^+^ NK cell inhibition assays. (**E**) Representative NKG2A^+^ NK cell activation measured by CD107a surface mobilization and expression of TNF-α upon co-culture with K562/HLA-E loaded with solvent control (no peptide) or the indicated peptides. Gated on viable CD3^-^ CD56^dim^ NKG2A^+^ NKG2C^-^ NK cells as in (D). (**F**) Summary of NKG2A^+^ NK cell activation measured by frequency of (left) CD107a, (middle) TNF-α, and (right) IFN-γ. Dots denote individual donors and bars mean (n = 6 donors in 3 independent experiments). Repeated-measures one-way ANOVA with Šídák’s multiple comparisons test (NS p ≥ 0.05 and **** p < 0.0001). (**G**) Cell surface HLA-E levels of K562/HLA-E cells loaded with either self-peptide alone or with peptide mixes consisting of 100 μM Nsp13_232-240_ variants combined with the indicated concentration of self-peptide. Dots and error bars denote mean±SEM (n = 8–9 independent experiments). Light grey and dark grey shaded area highlight concentrations selected for recombinant receptor binding assays (see Figs 4B–4D and S4H, respectively). (**H**) Summary of (left) HLA-E signals and (right) binding of recombinant NKG2A/CD94 upon loading with the indicated peptide mixes. Connected dots denote independent experiments (n = 4) and bars display mean. Repeated-measures one-way ANOVA with Šídák’s multiple comparisons test (NS p ≥ 0.05, * p < 0.05, and *** p < 0.001). (**I**) Peptides were mixed at a 1:9 ratio (self:viral) and K562/HLA-E cells were either treated with solvent control or loaded with 0.125, 0.5, 1.0, 100, 150, and 200 μM of peptide mixes, followed by co-culture with NK cells. Surface HLA-E levels on K562/HLA-E cells were determined in parallel. Each graph displays the CD107a response of NKG2A^+^ NK cells of an individual donor and the corresponding HLA-E signals detected on the target cells in parallel to the co-culture.
(PNG)

S5 Fig Hamming distances. Tabular summary of pairwise Hamming distances based on nucleotide differences in the indicated genomic sequences, as calculated relative to SARS-CoV-2 BA.5.
(PNG)

S1 Table Peptide sequences encoded by SARS-CoV-2 variants and related viruses. (XLSX)

S2 Table Antibodies used in this study. (DOCX)

S3 Table Synthetic peptides used in this study. (DOCX)
