# Supplementary figures and images for: Emerging mutation in SARS-CoV-2 facilitates escape from NK cell recognition and associates with enhanced viral fitness
Source: PLoS Pathog. 2024 Dec 9;20(12):e1012755. doi: 10.1371/journal.ppat.1012755 (PMC11658698; doi:10.1371/journal.ppat.1012755)

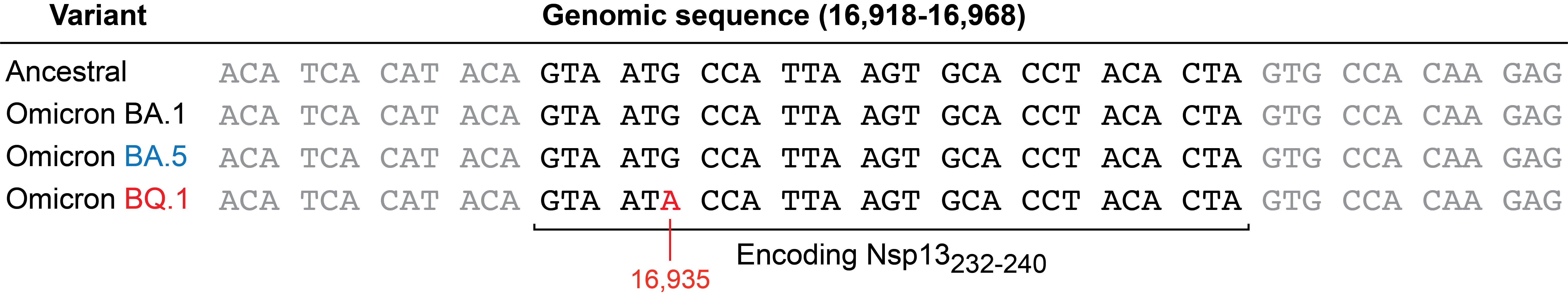

Supplement: S1 Fig — Genomic sequences of ancestral SARS-CoV-2 and Omicron sub-lineages around the Nsp13232-240-encoding sequence. (PNG) [file ppat.1012755.s001.png]

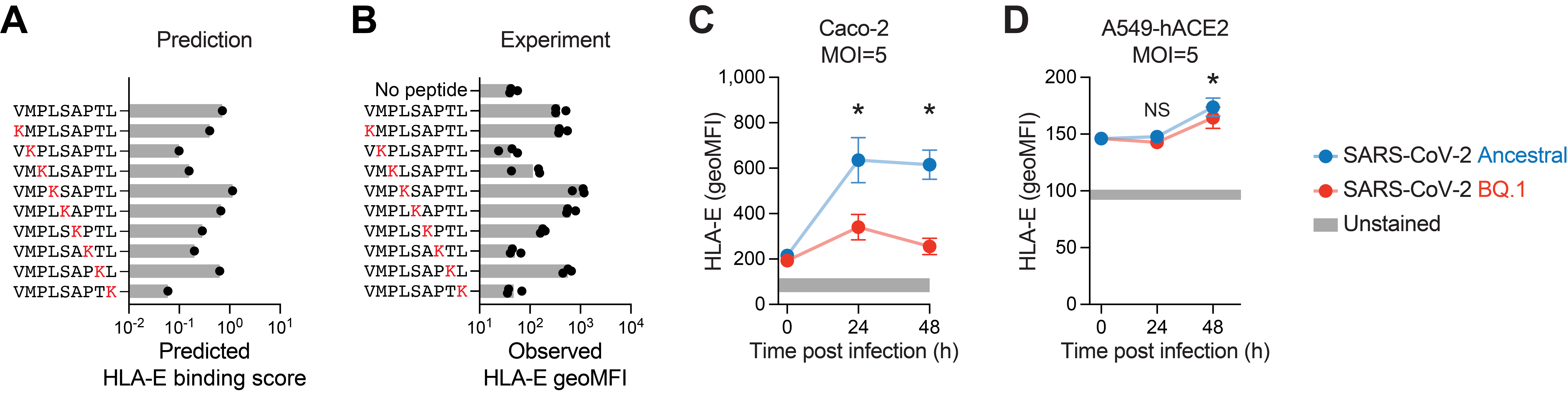

Supplement: S2 Fig — (A, B) Lysine scanning along the BA.5 Nsp13232-240 peptide sequence. (A) Predicted HLA-E binding scores and (B) measured surface HLA-E levels upon loading of K562/HLA-E cells with indicated peptides. Dots denote individual experiments and bars mean (n = 3 independent experiments). (C) Summary of surface HLA-E levels over time on Caco-2 cells following infection with MOI = 5. Dots denote means and error bars SEM (n = 4 infections in n = 2 independent experiments). Two-way ANOVA with Šídák’s multiple comparisons test (* p < 0.05). (D) Summary of surface HLA-E levels over time on A549-hACE2 cells following infection with MOI = 5. Dots denote means and error bars SD (n = 3 infections in n = 1 independent experiment). Two-way ANOVA with Šídák’s multiple comparisons test (NS p ≥ 0.05 and * p < 0.05). (PNG) [file ppat.1012755.s002.png]

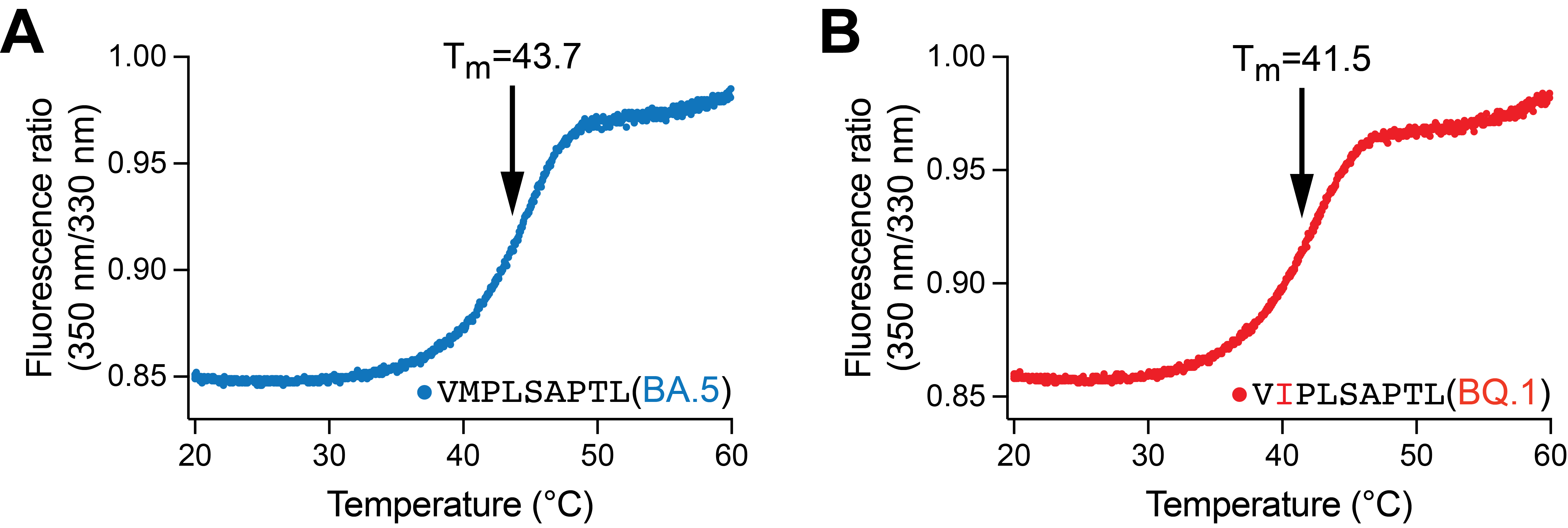

Supplement: S3 Fig — (A, B) Nano differential scanning fluorimetry analyses of (A) HLA-E*01:03/BA.5 Nsp13232-240 and (B) HLA-E*01:03/BQ.1 Nsp13232-240. Arrows denote inflection points, from which melting temperatures were derived. Representative curves of triplicate experiments are displayed. (PNG) [file ppat.1012755.s003.png]

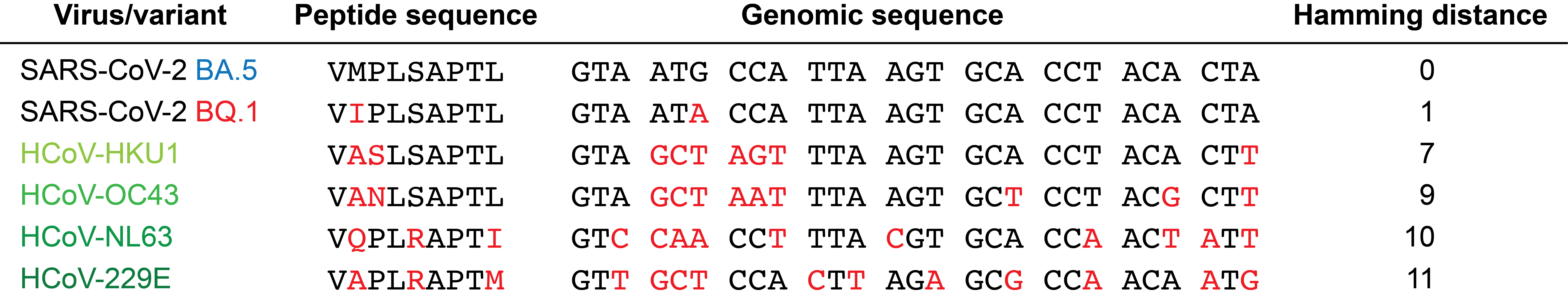

Supplement: S5 Fig — Tabular summary of pairwise Hamming distances based on nucleotide differences in the indicated genomic sequences, as calculated relative to SARS-CoV-2 BA.5. (PNG) [file ppat.1012755.s005.png]
