# Supplementary material for: Emerging mutation in SARS-CoV-2 facilitates escape from NK cell recognition and associates with enhanced viral fitness
Source: PLoS Pathog. 2024 Dec 9;20(12):e1012755. doi: 10.1371/journal.ppat.1012755 (PMC11658698; doi:10.1371/journal.ppat.1012755)
Supplement: S2 Table — (DOCX) [file ppat.1012755.s007.docx]

**S2 Table. Antibodies used in this study.**

| **Antibodies** | **Source** | **Identifier** |
| --- | --- | --- |
| Anti-human HLA-E-PE (3D12) | BioLegend | AB_1210774 |
| Anti-human CD107a-BUV395 (H4A3) | BD Biosciences | AB_2739073 |
| Anti-human TNF-α-BV650 (Mab11) | BioLegend | AB_2562741 |
| Anti-human IFN-γ-AlexaFluor700 (B27) | BD Biosciences | AB_396977 |
| Anti-human CD3-PE-Cy5 (UCHT1) | BioLegend | AB_314064 |
| Anti-human CD56-BUV737 (NCAM16.2) | BD Biosciences | AB_2860005 |
| Anti-human CD16-BV785 (3G8) | BioLegend | AB_2563803 |
| Anti-human CD57-PacificBlue (HNK-1) | BioLegend | AB_2562459 |
| Anti-human NKG2C-PE (REA205) | Miltenyi Biotec | AB_2751835 |
| Anti-human NKG2A-VioBrightFITC (REA110) | Miltenyi Biotec | AB_2726173 |
| Anti-human Fc-PE (polyclonal) | ThermoFisher | AB_465926 |
