# Supplementary material for: Emerging mutation in SARS-CoV-2 facilitates escape from NK cell recognition and associates with enhanced viral fitness
Source: PLoS Pathog. 2024 Dec 9;20(12):e1012755. doi: 10.1371/journal.ppat.1012755 (PMC11658698; doi:10.1371/journal.ppat.1012755)
Supplement: S3 Table — (DOCX) [file ppat.1012755.s008.docx]

**S3 Table. Synthetic peptides used in this study.**

| **Peptides** | **Source** | **Identifier** |
| --- | --- | --- |
| SARS-CoV-2 BA.5 Nsp13_232-240_  (VMPLSAPTL) | Peptides&Elephants, Genscript, JPT | N/A (custom order based on <https://www.iedb.org/epitope/70008>) |
| SARS-CoV-2 BQ.1 Nsp13_232-240_  (VIPLSAPTL) | Peptides&Elephants, Genscript, JPT | N/A (custom order, identified in this study) |
| HLA-C_3-11_  (VMAPRTLIL) | Peptides&Elephants, JPT | EP06244_5 (custom order based on <https://www.iedb.org/epitope/69921>) |
| HCoV-OC43 ORF1ab_5529-5537_  (VANLSAPTL) | Peptides&Elephants | N/A (custom order based on <https://www.iedb.org/epitope/1870818>) |
| HCoV-HKU1 ORF1ab_5597-5605_  (VASLSAPTL) | Peptides&Elephants | N/A (custom order based on <https://www.iedb.org/epitope/1870820>) |
| HCoV-229E ORF1ab_5233-5241_  (VAPLRAPTM) | Peptides&Elephants | N/A (custom order based on <https://www.iedb.org/epitope/1870819>) |
| HCoV-NL63 ORF1ab_5203-5211_  (VQPLRAPTI) | Peptides&Elephants | N/A (custom order based on <https://www.iedb.org/epitope/1870826>) |
| p1K of BA.5 Nsp13_232-240_  (KMPLSAPTL) | Peptides&Elephants | N/A (custom order for lysine scanning) |
| p2K of BA.5 Nsp13_232-240_  (VKPLSAPTL) | Peptides&Elephants | N/A (custom order for lysine scanning) |
| p3K of BA.5 Nsp13_232-240_  (VMKLSAPTL) | Peptides&Elephants | N/A (custom order for lysine scanning) |
| p4K of BA.5 Nsp13_232-240_  (VMPKSAPTL) | Peptides&Elephants | N/A (custom order for lysine scanning) |
| p5K of BA.5 Nsp13_232-240_  (VMPLKAPTL) | Peptides&Elephants | N/A (custom order for lysine scanning) |
| p6K of BA.5 Nsp13_232-240_  (VMPLSKPTL) | Peptides&Elephants | N/A (custom order for lysine scanning) |
| p7K of BA.5 Nsp13_232-240_  (VMPLSAKTL) | Peptides&Elephants | N/A (custom order for lysine scanning) |
| p8K of BA.5 Nsp13_232-240_  (VMPLSAPKL) | Peptides&Elephants | N/A (custom order for lysine scanning) |
| p9K of BA.5 Nsp13_232-240_  (VMPLSAPTK) | Peptides&Elephants | N/A (custom order for lysine scanning) |
